# The role of a checklist for assessing the quality of basic life support performance: an observational cohort study

**DOI:** 10.1186/s13049-018-0564-4

**Published:** 2018-11-16

**Authors:** Johanna van Dawen, Lina Vogt, Hanna Schröder, Rolf Rossaint, Lina Henze, Stefan K. Beckers, Saša Sopka

**Affiliations:** 1 Department of Orthopaedics and Trauma Surgery, Düren Hospital, Roonstraße 30, 52351 Düren, Germany; 2 Department of Anaesthesiology, University Hospital Aachen, Medical Faculty, RWTH Aachen University, Pauwelsstr. 30, 52074 Aachen, Germany; 3 Interdisciplinary Training Centre for Medical Education and Patient Safety – AIXTRA, Medical Faculty, RWTH Aachen University, Wendlingweg 2, 52074 Aachen, Germany; 4 Emergency Medical Service, Fire Department Aachen, Stolberger Str. 155, 52060 Aachen, Germany

**Keywords:** Basic life support (BLS), Cardiopulmonary resuscitation (CPR), External chest compression (ECC), Assessment checklist, Training, Medical education

## Abstract

**Background:**

Training lay rescuers in Basic Life Support (BLS) is essential to improve bystander cardiopulmonary resuscitation (CPR) rates; in addition, simple methods are needed to provide feedback on CPR performance. This study evaluated whether a simple observational checklist can be used by BLS instructors to adequately measure the quality of BLS performance as an alternative to other feedback devices.

**Methods:**

The BLS performances of 152 first-year medical students (aged 21.4 ± 3.9 years) were recorded on video, and objective data regarding the quality of the BLS were documented using Laerdal PC SkillReporting software. The performances were categorized according to quality. Ten BLS instructors observed the videos and completed a ten-point checklist based on the Cardiff Test of BLS (version 3.1) to assess the performances. The validity of the checklist was reviewed using interrater reliability as well as by comparing the checklist-based results with objective performance data.

**Results:**

Matching the checklist-based evaluation with the objective performance data revealed high levels of agreement for very good (82%) and overall insufficient (75%) performances. Regarding the checklist-based evaluation, interrater reliability depended on the checklist item; thus, some items were more easily identified correctly than others. The highest and lowest levels of agreement were observed for the items “undressed torso” and “complete release between compressions” (mean joint-probability 95 and 67%, respectively).

**Conclusions:**

The observational checklist adequately distinguished sufficient from insufficient BLS performances and offered an assessment of items not incorporated by SkillReporting software such as the initial assessment or undressing the chest. Although its usefulness was reduced for scaling intermediate performance groups, the checklist may be overall a useful rating tool in BLS-training if objective feedback devices are not available, for example, due to large groups of participants or limited training time.

## Background

Worldwide, about 17 million people per year die as a result of cardiovascular diseases [1]. In 25% of these cases, patients experience sudden cardiac death [2]. The high number of patients dying from sudden cardiac death as well as the low success rates of cardiopulmonary resuscitation (CPR) make sudden cardiac death a persisting predicament in patient care and public health [3–5].

In order to improve the currently insufficient implementation of CPR measures in out-of-hospital cardiac arrest (OHCA) patients in most countries, it is essential that lay rescuers receive efficient and extensive Basic Life Support (BLS) training [6]. Bystander-CPR is crucial to improving survival rates and neurological outcome in OHCA [7–9]. Furthermore, lay people trained in BLS are more willing to perform CPR in emergencies [7, 10]. As part of its latest guidelines, the European Resuscitation Council (ERC) recommends providing BLS training to every member of a community [7]. A 2013 study conducted the USA in 2013 revealed that financial factors are a main barrier for learning CPR in low-income environments and showed the necessity for low-cost or free BLS training in order to increase the number of lay people capable of performing CPR [11]. In addition, the implementation of BLS training may not proceed due to limited resources; for example, limited funds for CPR courses at schools, which underscores the importance of inexpensive but efficient learning strategies [12].

Several studies have estimated the cost-effectiveness of extensive CPR-training for laypersons since economic factors must be considered within health care systems, but further research is needed [13–15]. As feedback is an essential part of BLS training, several devices are available to assess CPR performance [16, 17]. For example, directive or audio feedback devices are recommended within the current ERC guidelines to improve the ability to perform CPR [7]. As such high-fidelity devices may not be available in low-income environments or financially weak surroundings, a simpler method of assessment is needed to provide feedback on CPR performance in these settings.In 1999, Graham et al. tested a scoring system based on simple observation as an inexpensive but effective method to assess CPR performance. The results suggested that an observation-based scoring system is an objective method to reflect the ability to perform BLS [18].

In the present study used a simple ten-point checklist modified after the Cardiff Test of BLS [19] to assess BLS performance recorded on video. The objective was to evaluate the checklist as a sufficient rating tool and an alternative instrument compared to SkillReporting software for CPR quality measurement using BLS training manikins.

## Methods

The data used for this study were acquired within the emergency medicine course for first-year medical students during the first three weeks of their curriculum at the Medical School of RWTH in Aachen, Germany in 2013. In total, 278 first-year medical students were included in the study.

Clinical experts in the field of emergency medicine and medical education designed the checklist based on the Cardiff test of BLS (version 3.1). The Cardiff test lists ten points associated with the quality of CPR based on the established ERC Guidelines.

The study was approved by the ethics committee of the Medical School of RWTH Aachen, Germany (EK- 100/12).

### Setting

Each participant was confronted with the same standardized scenario. They were expected to resuscitate a collapsed person represented by a BLS manikin (Resusci Anne™, Laerdal, Stavanger, Norway). None of the participants had received BLS training during their medical studies up to this point. Each student performance was recorded on videotape and performance data were obtained using Laerdal PC SkillReporting System Software (Version 2.4.1, Laerdal, Stavanger, Norway). The students were guided following a structured protocol and every student received exactly the same instructions. The scenario started equally every time, described as follows:

The participant was asked to enter a room in which a BLS manikin was lying on the floor with a zippered jacket covering the torso. No information about the scenario was provided in advance. The standardized text was read by the course instructor: *“Imagine you are witnessing a person collapsing right in front of you. The manikin represents this person. There is no one else nearby. Please take all measures you would take if the manikin was a real person. Keep going until you receive a signal to stop.”*

The performance was terminated 120 s after the first external chest compression (ECC). If the participant did not perform CPR, the scenario was stopped after 90 s. No further instructions were provided during the performance.

### Measurement and data acquisition

The performance data collected during the assessment was listed in a tabular form. The following three measuring criteria were identified as congruent to the ERC guidelines [7] and used in this study to determine the quality of CPR:

- ≥ 60% correct compression depth
- Average compression rate of 100–120 min^− 1^
- ≥ 60% compressions with complete release

Based on these criteria, the participant performances were assigned to four different categories based on the collected data and on how many of the criteria were met. The categories were color-coded and referred to a “traffic light classification.” An additional black category was defined for those who did not meet any of the criteria:

- Green: all three criteria were met
- Yellow: two of the three criteria were met
- Red: one of the criteria were met
- Black: none of the criteria we met

In order to compare the results regarding the quality of ECC assessed by either the Laerdal PC SkillReporting Software or by the checklist-based evaluation, the participant performances were recorded on video from the time that they entered the scenario until they were signaled to stop. Ten experienced BLS instructors were invited to rate the performance of every participant using the checklist. The raters were asked to use a nominal scale (1 = yes, 2 = no) to rate the criteria. The checklist rating criteria were defined as follows:

- Undressed torso
- Adequate minimum no-flow time (no longer than 2 s for two rescue breaths)
- Correct hand position
- Correct compression depth
- Correct compression rate
- Complete release between compressions
- Arms kept straight
- Vertical direction of compressions
- No delay to start CPR
- Compression-ventilation ratio of 30:2

The same standardized conditions were applied to the raters and the rating process. As a requirement, all raters had to be BLS instructors. The raters were instructed to observe each video for at least one minute and to evaluate the performance by means of the checklist. Soon after data collection, the video rating took place. None of the observers were involved in the training of the medical students whose performances were assessed since instructors assessing their own students reportedly tend to overestimate their competences [19]. The raters were informed in advance that the elements of the checklist were self-explanatory and no questions were answered during the evaluation process.

The Laerdal Rescue Anne with SkillReporting System Software assessed the following five items: correct hand position, correct compression depth and rate, complete release between compressions, and minimum no-flow time. After the study, all data were exported from the software.

At the time the study was performed, the current ERC Guidelines recommended an average compression rate of 100–120 min^− 1^, a compression depth of at least 50 mm, and complete chest recoil after each compression [20].

### Statistical methods

The interrater reliability was investigated for every item on the checklist by means of joint probability of agreement (in %) as well as Light’s Kappa (multi-rater version of Cohen’s kappa) in order to determine the agreement between the raters as a quality feature of the checklist as a rating tool. An average Kappa across all rater pairs was determined for every item of the checklist (mean Light’s kappa).

In order to examine the validity of the checklist items, the results of the checklist-based evaluation were compared to the performance data assessed by the Laerdal PC SkillReporting Software also using the joint probability of agreement and Light’s Kappa.

Sensitivity and specificity (in %) were calculated for the “correct compression rate,” “correct compression depth,” and “complete release between compressions” criteria. For sensitivity calculations, the number of performances correctly detected by the raters as matching the criteria was set as the “true positives.” To identify the true positive rate (sensitivity), the proportion of true positives was calculated among all performances that were classified as correct by the Laerdal PC SkillReporting System. Thus, the specificity or true negative rate was defined as the proportion of performances not matching the criteria which were correctly identified as such by the raters.

To compare the results of performance data and checklist-based evaluation in terms of the traffic light categories, the “correct compression rate”, “correct compression depth” and “complete release between compressions” checklist criteria were also used to assign the performance to one of the traffic light categories. For one of the criteria to apply, the mean checklist value across all raters for that item had to be less than 1.5 as a nominal scale was used to evaluate the performance (1 = yes, 2 = no). Using the classification by traffic lights for both performance data and checklist-based evaluation, it was possible to identify the number of performances that were assigned to the same traffic light category by both methods.

All statistical analyses were performed using IBM SPSS Statistics for Windows and Mac, version 23.0 (Armonk, NY: IBM Corp.).

## Results

### Study population

Of 278 potential participants, 152 were included in the study. All participants were first-year medical students at the medical faculty of RWTH Aachen University and had no relevant medical experience prior to their studies. Their mean age was 21.4 ± 3.9 years (range: 17–39). Among the participants, 67% were female, 26% were male, and 7% did not report their sex. One hundred and twenty-six subjects were excluded due to missing performance data, written consent, or video data.

### Observed endpoints

#### Performance data

The distributions of participants across the traffic light categories showed that only a small number of students achieved an overall adequate CPR performance by fulfilling all criteria (*n* = 11). The yellow category (fulfilling two out of three criteria) consisted of 52 (34.5%) participants. The largest group was represented by the red category (*n* = 79; 52,3%) consisting of participants whose performance matched only one criterion. Within the red group, 77 of 79 students achieved a complete release between compressions, two showed sufficient compression depth, and < 60% showed a complete release. The black group (none of the criteria) contained nine participants (Table 1).

**Table 1:** Performance data according to traffic light category

			Average compression rate(min^−1^)	Compressions with complete release(%)	Correct compression depth(%)
	Average compression rate of 100–120 min^−1^ and > 60% correct compression depth and > 60% compressions with complete release	N	11	11	11
		Mean	112.8	94.7	94.0
		SD	5.47	12.5	9.7
		Mini.	101	60.1	66.7
		Max.	120	100.0	100.0
	Average compression rate of 100–120 min^−1^ and > 60% compressions with complete release	N	28	28	28
		Mean	109.6	98.3	6.7
		SD	6.37	6.7	10.7
		Min.	100	64.7	0.0
		Max.	120	100.0	43.2
	> 60% compressions with complete release and > 60% correct compression depth	N	24	24	24
		Mean	102.8	98.0	85.4
		SD	33.5	8.0	13.5
		Min.	39	60.4	61.8
		Max.	153	100.0	100.0
	> 60% compressions with complete release	N	77	77	77
		Mean	94.8	99.3	10.7
		SD	31.1	02.5	16.1
		Min.	33	84.5	0.0
		Max.	187	100.0	57.7
	> 60% correct compression depth	N	2	2	2
		Mean	139.0	41.6	88.7
		SD	14.1	20.2	9.3
		Min.	129	27.3	82.1
		Max.	149	55.9	95.2
	Insufficient CPR performance in all categories	N	9	9	9
		Mean	61.4	12.9	19.4
		SD	73.6	19.4	33.1
		Min.	0	0.0	00.0
		Max.	160	54.6	95.2
	Total	N	151	151	151
		Mean	98.7	92.7	29.5
		SD	33.4	22.5	37.2
		Min.	0	0.0	0.0
		Max.	187	100.0	100.0

### Interrater reliability

There were considerable differences in the interrater reliability between the checklist items. While the items “undressed torso” (mean joint probability of agreement 94.9%; mean Kappa 0.866) and “compression-ventilation ratio of 30:2” (mean joint probability of agreement 85.3%; mean Kappa 0.630) had equal explicit measurements across all raters, the item “complete release between compressions” (mean joint probability of agreement 67.2%; mean Kappa 0.295) showed great variation (Table 2).

**Table 2:** Interrater reliability for all subjects (*n* = 152)

	Mean joint probability of agreement (%)	Mean Light’s Kappa
Undressed torso	94.9 (±1.8)	0.87 (±0.11)
Compression-ventilation ratio of 30:2	85.3 (±2.7)	0.63 (±0.05)
No delay in starting CPR	76.8 (±4.6)	0.45 (±0.11)
Correct compression rate	76.6 (±5.4)	0.49 (±0.07)
Arms kept straight	76.3 (±7.1)	0.46 (±0.11)
Correct compression depth	75.3 (±3.4)	0.49 (±0.04)
Vertical direction of compressions	74.2 (±4.6)	0.44 (±0.09)
Minimum no-flow time	73.6 (±9.8)	0.42 (±0.16)
Correct hand position	72.2 (±2.7)	0.47 (±0.08)
Complete release between compressions	67.2 (±4.5)	0.30 (±0.06)

### Matching rater evaluations and performance data

Comparison of the checklist-based evaluation by the raters with the performance data obtained by the Laerdal PC SkillReporting software revealed differences in the descriptive values of mean Light’s Kappa between items. The joint probabilities of agreement (%) between raters and software were close for the items (Table 3).

**Table 3:** Agreement between performance data and checklist-based evaluation for all subjects (*n* = 152)

	Mean joint probability of agreement (%)	Mean Light’s Kappa	Sensitivity			Specificity		
			*n*	Range(%)	Mean(%)	*n*	Range(%)	Mean(%)
Correct compression rate	72.6 (±7.3)	0.41 (±0.14)	39	48.7–94.9	77.5	111	55.0–91.0	71.0
Correct compression depth	70.1 (±6.4)	0.35 (±0.14)	37	50.0–94.3	74.3	113	50.9–78.9	68.8
Complete release between compressions	67.7 (±12.7)	0.15 (±0.11)	140	44.8–83.0	67.6	10	50.0–88.9	68.5

Across all categories, the item “correct compression rate” showed the highest agreement between performance data and checklist-based evaluation (mean joint probability of agreement 72.6%; mean Kappa 0.41). The largest range was observed for the item “complete release between compressions” (mean joint probability of agreement 67.7%; range 47.3–82.7%).

The sensitivity and specificity of the different checklist items were also highest for the item “correct compression rate”, while the item “complete release between compressions” had the lowest sensitivity and specificity. Generally, the sensitivity was slightly higher than the specificity for all items (Table 3).

Regarding the item “compression rate”, a compression rate lower than 100 min^− 1^ was more often correctly identified as wrong (sensitivity mean: 90.0%; range: 77.1–97.1%) than a compression rate higher than 120 min^− 1^ (sensitivity mean: 38.4%; range: 17.1–87.8%).

Concerning the traffic light classification, out of all performances defined as “green” by the performance data (*n* = 11), 81.8% (*n* = 9) of the performances were also assigned to the green category using the checklist-based evaluation. In terms of the black category, 75.0% (*n* = 6) of the participants were allocated correctly using the checklist data.

In contrast, within the yellow category (*n* = 52), only 50.0% (*n* = 26) matched that category according to the checklist-based evaluation data. Within the red category (*n* = 79), the result was even lower (35.4%, *n* = 28) (Table 4).

**Table 4:** Distributions of traffic light categories by checklist-based evaluation within traffic light categories of performance data

Traffic light category by performance data		Traffic light categoryby checklist-based evaluation				Total
			
	*n*	9	1	1	0	11
	Number of performances within category (%)	81.8	9.1	9.1	0.0	100.0
	*n*	17	19	11	5	52
	Number of performances within category (%)	32.7	36.5	21.2	9.6	100.0
	*n*	9	24	23	23	79
	Number of performances within category (%)	11.4	30.4	29.1	29.1	100.0
	*n*	1	0	1	6	8
	Number of performances within category (%)	12.5	0.0	12.5	75.0	100.0

### Discussion

This observational cohort study evaluated whether an observational checklist was an adequate assessment tool for BLS instructors to estimate the quality of a CPR performance.

The main result was that the use of the observational checklist appropriately distinguished between overall good and overall insufficient performances. This was demonstrated by the allocation of the participants to the green and the black categories based on the checklist in accordance with the objective performance data-based distribution. Regarding all adequate CPR performances (as defined by the skill reporter), 81.8% were identified as such by the checklist-based evaluation. In contrast, the low agreement between the performance data-based and the checklist-based allocation regarding the yellow and the red categories suggests that the use of the checklist is not suitable to differentiate between mediocre performances.

The study further indicated that crucial elements of CPR, such as minimum delay to start CPR, correct compression-ventilation ratio, and undressing the torso, were accurately assessable by simple observation, which is shown by the high interrater reliability. However, these aspects cannot be recorded by skill reporter systems. The low interrater reliability for complete release between compressions suggests that this item is not easily accurately identified by simple observation and benefits from SkillReporting software.

Furthermore, the comparison of the sensitivity and specificity suggests that correct performance was easier for the raters to identify, whereas incorrect performance was more difficult to detect.

Graham et al. also suggested that a simple scoring system is a valid method to assess CPR performances. Students were evaluated based on a 10-point checklist and were assigned penalty points when the element was performed incorrectly. The scoring system differed between minor, moderate, and serious errors in the number of penalty points. The participants were assigned only to “pass” or “fail” categories without distinguishing the quality of CPR. Their study presented observed and performance data but, unlike the present study, did not compare their results to objectively obtained data from SkillReporting software [18].

In a more recent study, Kim et al. also used a checklist-based evaluation to assess BLS performances in medical students. The checklist consisted of 11 items representing the BLS algorithm such as initial patient assessment and calling for help, as well as performing CPR, including compression-ventilation ratio and correct hand position as independent items, whereas compression rate and depth was a single item. The participants were assessed as “correct” or “incorrect” for each item and graded on a scale from 1 to 5 for the whole performance. Within their study, the assessment by BLS instructors was compared to self-assessment by the students, both using the same checklist. Interestingly, the analysis showed no significant differences between tutor and self-assessments [21].

Whether the checklist used within our study could also be used for adequate self-assessment by medical students or laypersons is a topic for further study. Additionally, the influence of the implied setting on the applicability of the checklist, for example, in different study populations, requires further investigation.

If the checklist-based evaluation was used to assess real cases of CPR in OHCA, it could be interesting to investigate whether the raters would evaluate performances differently if they were aware of the patient outcomes.

Another point of interest was how the raters are influenced while evaluating a CPR performance by means of the checklist. It is possible that a good performance for most items on the checklist might lead the rater to be more indulgent with an inaccurate performance for other items. In addition, an altogether poor performance could bias the rater to more negatively evaluate each criterion.

A low-tech feedback device such as the checklist used in the current study might be useful in the implementation of CPR training for large groups such as school classes, where high-fidelity manikins might not be available, for example, due to limited funds. Training schoolchildren in CPR is a highly effective method to improve bystander CPR and patient outcome in OHCA [22–24].

### Limitations

Due to the recording of the performance from only one perspective, some of the video data could not be assessed by the raters. This is a limitation to use the checklist to evaluate performances, but only if the rater is unable to directly observe the performance.

Most of the performances were inadequate because untrained lay persons were observed in this study. Having mainly negative performances makes false positive evaluations carry more weight than false negative ones. Due to that fact, both sensitivity and specificity have been calculated.

In terms of the traffic light categories, compression rates not between 100 and 120 min^− 1^ were identified as wrong based on the ERC guidelines. Thus, a compression rate of 121 was valued the same as a rate of 0. This example of two different performances not matching the previously determined criteria cannot have equally negative effects on patient outcome. In this particular case, the developed checklist might allow users to distinguish between the two since it is slightly more inaccurate and accepts performances with compression rates very close to the recommended range while also detecting inadequate compression rates with a high specificity.

## Conclusions

A simple observational checklist can be used to assess BLS quality and identify sufficient and insufficient performances. In order to provide more detailed feedback concerning CPR, skill feedback devices may be useful in addition to the checklist. The checklist is a valuable assessment tool if high-tech feedback devices are not available or useful; for example, due to high numbers of participants in training groups or limited training time.

## Abbreviations

BLS: Basic life support
CPR: Cardiopulmonary resuscitation
ECC: External chest compression
OHCA: Out-of-hospital cardiac arrest
